# A Super-Sensitive Voltammetric Procedure for the Determination of Pb(II) Ions in Natural Waters Using an Environmentally Friendly Microelectrode

**DOI:** 10.3390/ma18215022

**Published:** 2025-11-04

**Authors:** Malgorzata Grabarczyk, Wieslawa Cwikla-Bundyra

**Affiliations:** Department of Analytical Chemistry, Institute of Chemical Sciences, Faculty of Chemistry, Maria Curie-Sklodowska University, 20-031 Lublin, Poland; wieslawa.cwikla-bundyra@mail.umcs.pl

**Keywords:** lead(II), determination, anodic stripping voltammetry, microelectrode, environmental waters

## Abstract

A versatile voltammetric procedure for quantitative determination of Pb(II) directly in environmental water samples has been proposed. Differential pulse technique in the variant of anodic stripping voltammetry was applied to study Pb(II) at a solid bismuth microelectrode (SBiµE). The proposed procedure was tested using model solutions containing 0.1 mol L^−1^ acetate buffer (pH = 3.4) and 5 × 10^−9^ mol L^−1^ Pb(II). Under optimized measurement conditions, i.e., activation potential and time −2.5 V and 30 s, respectively, and accumulation potential and time −1.4 V and 30 s, respectively, a linearity range of 1 × 10^−10^–3 × 10^−8^ mol L^−1^, a detection limit of 3.4 × 10^−11^ mol L^−1^ and a relative standard deviation of 3.1% were obtained. The applicability of the developed procedure was confirmed by direct analysis of environmental waters, such as water from the Bystrzyca River and water from the Baltic Sea.

## 1. Introduction

With the development of industry, increasing environmental pollution by lead has been observed for many years, mainly related to anthropogenic human activities, causing increased lead concentrations in soils, waters, and atmospheric dust. Lead has many applications in the construction industry, as an alloy additive, sound, vibration, and radiation barriers, the largest use of lead in the early 21st century; however, it is in lead-acid batteries. Significant amounts of lead are introduced into the environment by hunters, estimated at thousands of tons per year of lead shot introduced into soils. Of course, natural sources of lead contamination of the natural environment should not be forgotten either, which are mainly dust and gases from volcanic eruptions and forest fires [1,2,3,4,5,6,7,8]. As lead is among the elements that are highly toxic and easily transformed in the environment, it is one of the heavy metal ions most commonly determined in various environmental samples. Most countries set limit values for lead content, but according to some experts, there is no safe level of lead in the environment; exposure to any amount is considered harmful. Therefore, there is a constant demand for dedicated procedures employing different analytical techniques.

Reliable and inexpensive analytical methods for the detection and determination of even trace concentrations of Pb(II) ions are based, among others, on stripping voltammetry. While this technique allows for the determination of various types of analytes, it is often dedicated to metal ions, among others, due to the possibility of obtaining direct speciation, taking into account the oxidation state, or the assessment of the content of the labile form of a given ion. This is also the case for the determination of Pb(II) ions directly in aqueous environmental samples using the stripping voltammetry technique, a variant of which is mainly anodic stripping voltammetry (ASV) [9,10,11,12], but works using adsorption stripping voltammetry (AdSV) [13,14,15] can also be found.

A strategic choice for the voltammetric measurement is the choice of working electrode material. In the early stages of the development of voltammetry, mercury electrodes, the hanging drop mercury electrode (HMDE), and the film mercury electrode (MFE) were commonly used as working electrodes. Mercury electrodes have many advantages; they are the closest to a perfectly polarized electrode due to their almost perfectly smooth, pure mercury surface, which is a liquid at room temperature. Mercury, as a noble metal, behaves inertly to most solutions and allows operation at very negative potential values [16,17,18,19,20]. Also, for the determination of Pb(II) by voltammetry, the first procedures described in the literature mainly used mercury electrodes. And, given the advantages of mercury electrodes mentioned above, in recent years, one can find in the literature voltammetric procedures for the determination of Pb(II) using as working electrodes modern designs and modifications containing mercury [21,22,23,24,25,26], including the mercury-modified screen-printed electrodes, which have attracted considerable interest in recent years [27,28,29,30]. Nevertheless, with the growing awareness of mercury toxicity, more and more procedures based on environmentally safer electrode materials have started to appear in the literature in recent years. In the case of Pb(II) determination, modified or non-modified carbon materials, carbon pastes, and carbon screen-printed electrodes can be mentioned [31,32,33,34,35,36,37].

Another significant part of the work has focused on the use of various types of bismuth-based electrodes for Pb(II) determination. The development of bismuth electrodes began in 2000, when Joseph Wang et al. first proposed and used a bismuth film electrode as a working electrode for voltammetric measurements [38]. Bismuth shows very little toxicity and is environmentally friendly, so it can provide an alternative to mercury electrodes, which dominate voltammetric measurements. The bismuth film can be prepared by in situ electrolysis. In this case, the bismuth salt must be introduced into the solution to be analyzed, and the film formed by applying an appropriate potential to the substrate electrode, or ex situ, where the bismuth film is applied from a separate bismuth salt solution. For the determination of Pb(II), in addition to the classical bismuth membrane electrodes, various novel bismuth-based design solutions have also been proposed. In recent years, bismuth has been combined with various materials such as carbon nanomaterials, conductive polymer, and metal nanoparticles. This can include electrodes such as in situ plated bismuth film screen-printed gold electrode [39], in situ plated bismuth film graphite carbon paste electrode [40], in situ plated bismuth film multiwalled carbon nanotube Nafion composite modified glassy carbon electrode [41], nanoporous bismuth electrode [42], nanocomposite functionalized multiwalled carbon nanotubes with polypyrrole film and bismuth particles carbon paste electrode [43], polymer/bismuth film modified glass carbon electrode [44], gold nanoparticle-graphene-cysteine composite modified bismuth film glassy carbon electrode [45], bismuth oxycarbide Nafion modified glassy carbon electrode [46], carbon paste electrode with BiOCl (bulk modifier) [47], (BiO)_2_CO_3_ nanoparticles@SWCNT-Nafion/GCE [48], bismuth (Bi)-biopolymer (chitosan) nanocomposite screen-printed carbon electrode [49], bismuth oxide surface-decorated nanoporous bismuth [50], bismuth-film electrode supported on pencil-lead graphite [51], bismuth film glassy carbon electrode with Zn mediator [52].

Depending on the size, among the working electrodes, we can distinguish micro and macro electrodes. Macro electrodes are definitely more frequently used in practice, including the bismuth electrodes described above, used for the determination of Pb(II) ions. Microelectrodes in voltammetry are characterized primarily by their high sensitivity, and thanks to their small size, they generate minimal capacitive currents, which results in a better signal-to-noise ratio; additionally, microelectrodes allow miniaturization of instruments. Therefore, a solid bismuth microelectrode (SBiµE) with Ø = 25 μm was constructed using very small amounts of solid bismuth. This electrode was first used to determine folic acid, and its exact design was described in a paper devoted to this procedure [53]. To date, this solid bismuth microelectrode (Ø = 25 μm) has been successfully used for the determination of analytes such as Sn(II), V(V), W(VI), Tl(I), Ga(III), Se(IV) allowing the development of environmentally friendly procedures with low detection limits [54,55,56,57,58,59]. An additional advantage of such an electrode is that Bi(III) ions do not need to be added to the solution, which is necessary for the formation of film bismuth electrodes, thus making the solid bismuth microelectrode part of green chemistry and making it environmentally friendly. Recently, a solid bismuth microelectrode array placed in a single housing has been described in the literature. This was used for the simultaneous determination of Pb and Cd using the square wave method, achieving good peak separation [60]. The aim of our research was to obtain the lowest possible detection limit for Pb(II) using an environmentally friendly, reusable bismuth microelectrode. For this purpose, SBiµE with a diameter of 25 μm was used, which, in combination with the differential pulse technique, allowed us to obtain a very low detection limit of 3.4 × 10^−11^ mol L^−1^.

## 2. Materials and Methods

### 2.1. Instrumentation Equipment

Differential pulse anodic stripping voltammetry (DPASV) was performed in the conventional three-electrode cell with an Autolab PGSTAT 10 analyzer. The conventional three-electrode system included a SBiµE (Ø = 25 μm) as the working electrode, an Ag/AgCl reference electrode (in saturated NaCl), and a platinum counter electrode were used as the reference electrode and auxiliary electrode. All electrochemical measurements were carried out in a static cell after stirring with a magnetic stirrer at room temperature.

A glass capillary 25 μm in diameter filled with molten metallic bismuth was used to construct a solid bismuth microelectrode. Such a capillary was placed in a housing made of PEEK, and carbon black and copper wire were used to achieve electrical contact. Each measurement day, the microelectrode was polished on silicon carbide paper 2500. And then, after washing with distilled water, it was placed in an ultrasonic bath (Sonic-3, Polsonic, Warsaw, Poland) for a period of 30 s to remove any residual polishing material.

### 2.2. Reagents

An acetate buffer (1 mol L^−1^) was prepared from Suprapur CH_3_COOH and NaOH obtained from Merck. Other reagents were obtained from POCh, Poland. All solutions were made using triply distilled water. Working solutions of Pb(II) were prepared every day by dilution of 1 g L^−1^ lead standard solution from Merck (Darmstadt, Germany).

### 2.3. Procedure for the DPASV Analysis

Metallic bismuth can passivate in air, becoming covered by a layer of bismuth oxide (Bi_2_O_3_), which adversely affects the concentration efficiency (in our case of lead) on the solid surface of the bismuth microelectrode. Therefore, each voltammetric measurement started with an electrochemical activation of the electrode surface by applying a potential of −2.5 V to it for 30 s. During this time, the possibly formed bismuth oxide is reduced to a metallic form, and the activated electrode surface allows efficient lead accumulation. Only after this stage, a potential of −1.4 V is applied to the electrode also for 30 s, during which stage lead ions accumulate on the electrode in metallic form. Then, after a rest period of 5 s, a differential pulse stripping voltammogram was recorded in the equilibrium solution by applying a positive-going potential scan from −0.8 to −0.35 V. At this stage, Pb(0) is oxidized back to Pb(II). The instrumental parameters of the differential pulse voltammetric measurement were as follows: scan rate 40 mV s^−1^, a pulse height of 50 mV, modulation time 0.005 s, and interval time 0.1 s.

All voltammetric measurements were carried out from a solution of 0.1 mol L^−1^ acetate buffer, pH = 3.4, which acted as the supporting electrolyte.

## 3. Results

### 3.1. Optimization for the Detection of Pb(II) at SBiµE

In order to obtain the best voltammetric behavior of the SBiµE towards Pb(II), a number of optimization experiments were carried out. The following parameters were investigated: the composition of the supporting electrolyte, the potential and activation time of the SBiµE surface, and the potential and accumulation time of the lead.

#### 3.1.1. Composition of the Supporting Electrolyte

**Figure 1:**
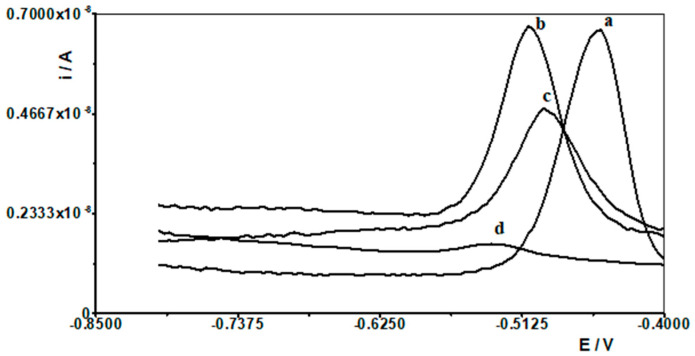
Differential pulse voltammograms obtained in the course of the 5 × 10^−9^ mol L^−1^ Pb(II) determination in various supporting electrolytes: (a) CH_3_COOH, (b) HNO_3_, (c) HClO_4,_ and (d) H_2_SO_4_.

**Figure 2:**
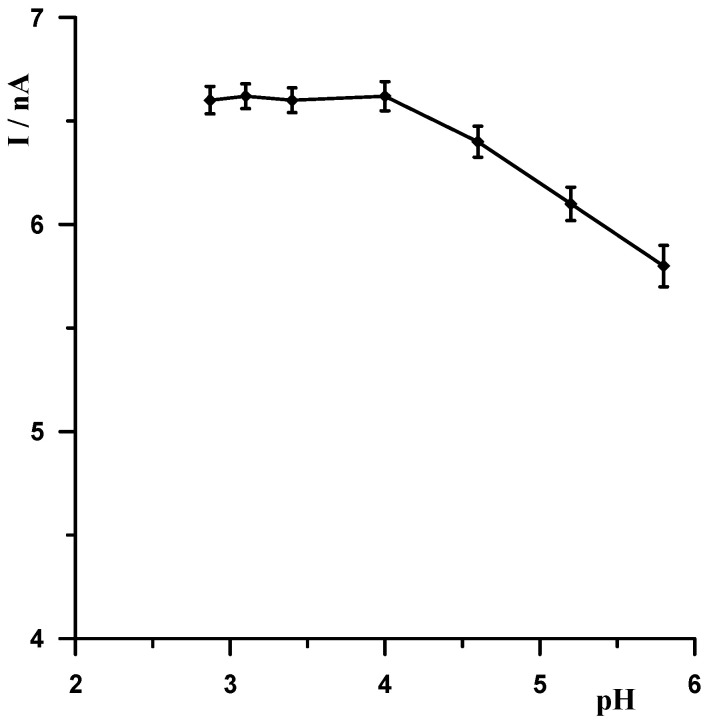
The influence of the pH of the supporting electrolyte on the Pb(II) peak current. Concentration of Pb(II) 5 × 10^−9^ mol L^−1^. Activation conditions −2.5 V and 30 s, and accumulation conditions −1.4 V and 30 s.

When selecting the supporting electrolyte, it was known from the outset that it must be acidic due to the fact that in a neutral and alkaline environment, bismuth undergoes a hydrolysis process, which cannot be allowed to occur. Therefore, the following acids were chosen for the study: HNO_3_, HClO_4_, H_2_SO_4_, and CH_3_COOH. Figure 1 shows example voltammograms for 5 × 10^−9^ Pb(II) recorded in solutions of various acids of 0.1 mol L^−1^ used as supporting electrolytes. As can be seen, the best signal was obtained for acetic acid. The next step was to check and compare the analytical lead signal recorded in acetic acid and acetate buffers. A series of acetate buffers with pH ranging from 3.1 to 5.8 was prepared for the study. Figure 2 shows how the lead signal changes as a function of the pH of the supporting electrolyte. As can be seen, the lead signal remains constant up to pH 4 and then gradually decreases. Therefore, an acetate buffer of pH = 3.4 was chosen as the most optimal for further studies. The magnitude of the lead peak is similar in acetic acid and in the acetate buffer at pH = 3.4; however, as the buffer prevents significant pH changes when a real sample of different pH is added, the choice of buffer as the base electrolyte ensures better stability of the signal.

#### 3.1.2. Conditions for Electrode Activation

**Figure 3:**
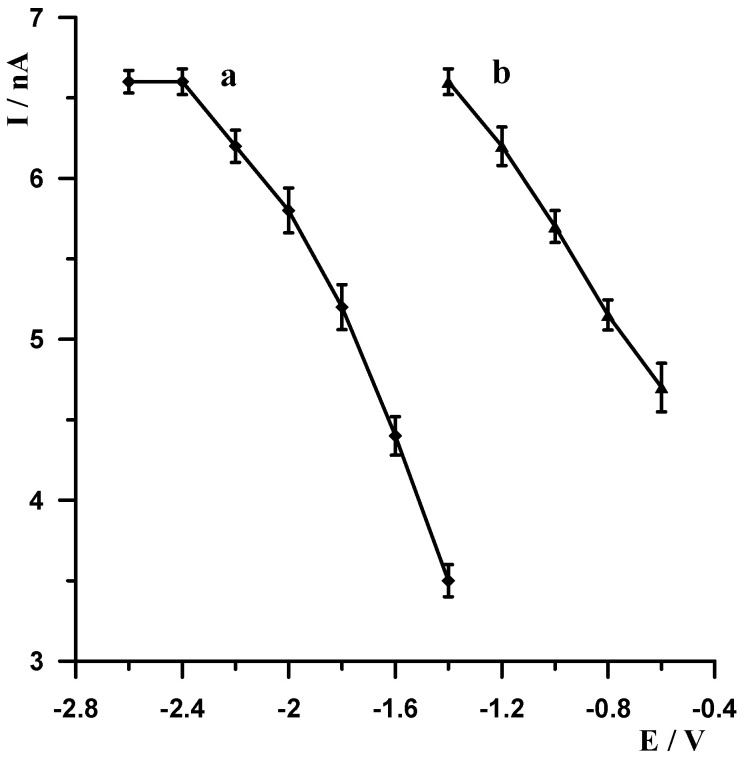
The influence of the activation potential for fixed accumulation potential −1.4 V (a) and accumulation potential for fixed activation potential −2.5 V (b) on the Pb(II) peak current. Concentration of Pb(II) 5 × 10^−9^ mol L^−1^, supporting electrolyte 0.1 mol L^−1^ acetate buffer (pH = 3.4). Activation and accumulation time 30 s.

**Figure 4:**
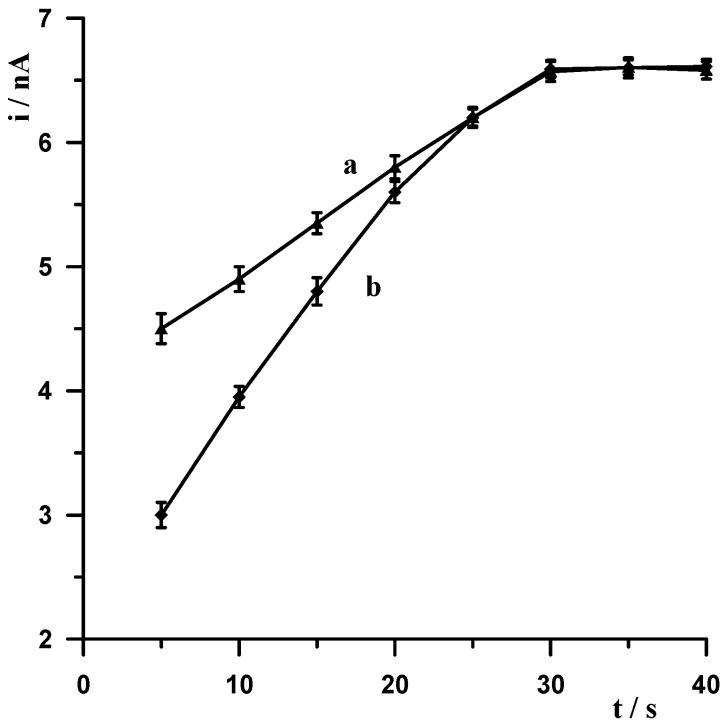
The influence of the accumulation time for fixed activation time 30 s (a) and activation time for fixed accumulation time 30 s (b) on the Pb(II) peak current. Concentration of Pb(II) 5 × 10^−9^ mol L^−1^, supporting electrolyte 0.1 mol L^−1^ acetate buffer (pH = 3.4). Activation and accumulation time was −2.5 V and −1.4 V.

As already mentioned, metallic bismuth readily undergoes passivation in air, becoming covered by a layer of bismuth oxide (Bi_2_O_3_), which adversely affects the accumulation efficiency of the analyte being determined on the surface of the bismuth electrode. A simple yet very effective way to eliminate passivation is electrochemical activation of the electrode. Therefore, each voltammetric measurement started with the application of a very negative potential to the electrode in order to remove the bismuth oxide layer by electrochemical reduction. The following experiments were carried out to select the most optimal potential value and duration. A solution of 0.1 mol L^−1^ acetate buffer containing 5 × 10^−9^ mol L^−1^ Pb(II) was prepared, and a series of measurements was carried out by varying the activation potential for 30 s at constant lead accumulation conditions of −1.4 V for 30 s. The activation potential was varied from −2.6 V to −1.4 V. The results obtained are shown in Figure 3 (curve a). A series of measurements was then carried out in which the activation potential was held constant at −2.5 V and the duration was varied from 5 to 40 s. As expected, with increasing activation time, the size of the lead peak increased, the peak current increased with increasing time up to 30 s, and then remained constant. The results obtained are shown in Figure 4 (curve b). After an in-depth analysis of the effect of activation potential and duration on lead signal size, −2.5 V and 30 s were chosen as optimum.

#### 3.1.3. Conditions for Lead Accumulation

In stripping voltammetry, the limit of quantification is significantly lowered compared to classical voltammetry, thanks to the introduction of a concentration step for the analyte being determined. In the procedure described in this manuscript, the concentration step involves the decrease in Pb(II) ions to the metallic form. The aim of our subsequent experiments was to select the accumulation potential and time. Measurements were carried out for a solution of 0.1 mol L^−1^ of acetate buffer containing 5 × 10^−9^ Pb(II). The measurement conditions were as follows: −2.5 V and 30 s electrode activation, accumulation for a potential varying from −1.4 V to −0.6 V for 30 s. For each potential, the voltammogram was recorded, and the results obtained are shown in Figure 3 (curve b). Identical measurements were carried out using a constant accumulation potential of −1.4 V and varying its duration from 5 to 40 s, and the results show that the peak increased as the accumulation time increased to 30 s. The results obtained are shown in Figure 4 (curve a). Based on the results obtained, a potential and time of −1.4 V and 30 s, respectively, were selected as the most optimal accumulation conditions.

### 3.2. Analytical Performance and Discussion

**Figure 5:**
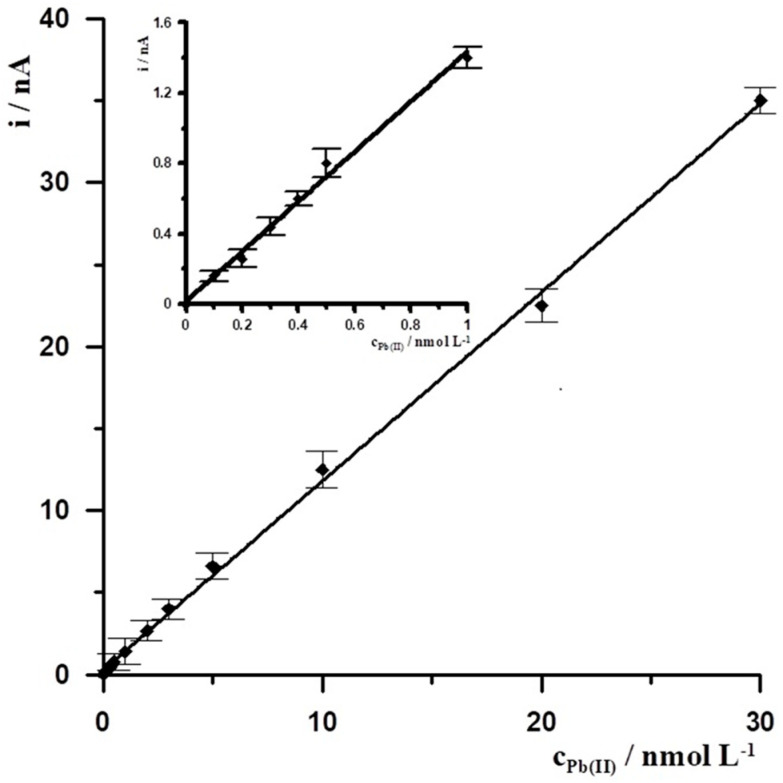
The Pb(II) calibration graph for activation conditions of −2.5 V and 30 s, and accumulation conditions of −1.4 V and 30 s. Supporting electrolyte 0.1 mol L^−1^ acetate buffer (pH = 3.4). Insert the calibration curve for the concentration range from 1 × 10^−10^ to 1 × 10^−9^ mol L^−1^.

Under the optimal experimental conditions, SBiµE was applied to the successive determination of Pb(II) in 0.1 mol L^−1^ acetate buffer (pH = 3.4) by DPASV. The DPASV response signal for different Pb(II) concentrations was determined, and a calibration curve plot was prepared based on it. The Pb(II) calibration graph for an activation conditions −2.5 V and 30 s and accumulation conditions −1.4 V and 30 s was linear in the range from 1 × 10^−10^ to 3 × 10^−8^ mol L^−1^ and obeyed the equation y = 1.16x + 0.26, where y and x are the peak current (nA) and Pb(II) concentration (nmol L^−1^), respectively, with the linear correlation coefficient r = 0.9993, the calibration curve is presented in Figure 5. The relative standard deviation from five determinations of Pb(II) at a concentration of 5 × 10^−10^ mol L^−1^ was 3.1%. The detection limit estimated from three times the standard deviation of low Pb(II) concentration was about 3.4 × 10^−11^ mol L^−1^.

Table 1 presents the analytical performance of Pb(II) determination by anodic stripping voltammetry using electrodes based on bismuth or combinations of bismuth with various materials. This summary was prepared on the basis of procedures described in recent years in the international scientific publication. As can be seen, the detection limits obtained in these procedures vary widely in concentration from 3.4 × 10^−11^ to 5.6 × 10^−8^ mol L^−1^, with the lowest detection limit obtained in the procedure described in this manuscript. This is a major advantage of this procedure, as such a low detection limit makes it possible to perform determinations on a wide range of samples with different lead contents, making this procedure very universal. The determination of such low concentrations allows, in the case of samples with a complex matrix, to perform determinations after their multiple dilution, thus minimizing the interfering effect of the matrix on the analytical signal. When analyzing the data compiled in the table, it is shown that the linearity range most often covers two orders of magnitude, and in a few cases, one order of magnitude. The procedure we propose provides a linearity range of more than two orders of magnitude, which also makes it more convenient to use in practice. Another key advantage of the electrode proposed in our work is the fact that its preparation is simple compared to most of the electrodes listed in the table, and the electrode itself is reusable and can be used for measurements for years.

### 3.3. Repeatability, Reproducibility, and Long-Term Stability of SBiµE

In order to determine the repeatability of the solid bismuth microelectrode, a series of six measurements was performed for two different solutions containing 5 × 10^−10^ and 5 × 10^−9^ mol L^−1^ Pb(II). Based on the peaks obtained on the voltammetric curves, the RSD was determined to be 3.3 and 2.5%. The reproducibility of the solid bismuth microelectrode was also determined by performing a series of measurements over six consecutive days with the same Pb(II) concentrations as in the repeatability calculation. The RSD obtained was 3.5 and 2.8%. In addition, the long-term stability of the solid bismuth microelectrode was determined by comparing the lead signals obtained at intervals of 6 months, and it was found that the difference in their magnitude was less than 3.4%.

### 3.4. The Influence of Lanthanides as Interferents

In recent decades, there has been a dynamic increase in the extraction and use of lanthanides, which have many unique physical and chemical properties. Lanthanides are a key raw material in various industrial sectors, especially in the manufacture of high-precision new technologies. They are used in pharmacology and medicine, in plant and animal production to accelerate the growth and fattening of farm animals, and as fertilizers, as they have a beneficial effect on plant growth [62]. The widespread use of lanthanides in various industries and in agriculture leads to a steady increase in the concentrations of these elements in the environment. Many publications devoted to the determination of Pb(II) ions using bismuth as an electrode material by the ASV method have investigated the influence of many different ions, mainly metals, but no study has investigated the influence of lanthanides, which are increasingly common as environmental pollutants.

**Figure 6:**
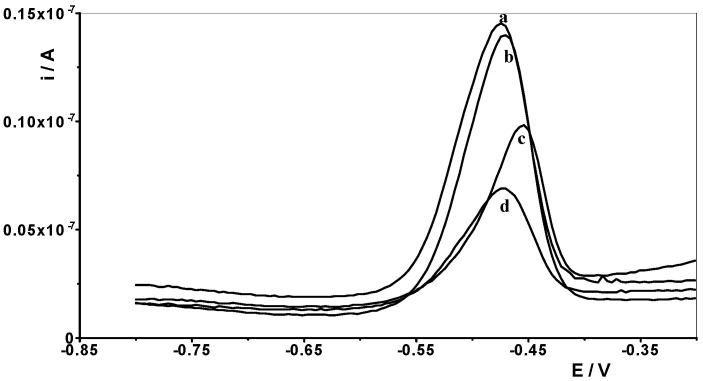
Differential pulse voltammograms obtained for 1 × 10^−8^ mol L^−1^ Pb(II) in the presence of the following La(III) concentrations: 0 (a); 1 × 10^−5^ mol L^−1^ (b), 5 × 10^−4^ mol L^−1^ (c), and 1 × 10^−4^ mol L^−1^ (d).

Therefore, one of the goals of our research was to determine the effect of selected lanthanides on the analytical signal of Pb(II). The following lanthanides were selected for testing: La(III), Ce(III), Pr(III), Nd(III), Eu(III), Gd(III), Dy(III), and Yb(III). A synthetic sample containing a constant concentration of 1 × 10^−8^ mol L^−1^ Pb(II) and a supporting electrolyte of 0.1 mol acetate buffer was prepared, and measurements were performed for it in accordance with the selected conditions, i.e., activation potential and time of −2.5 V and 30 s, respectively, and accumulation potential and time of −1.4 V and 30 s, respectively. Then, an increasing concentration of the selected lanthanide was added to the solution in the range of 1 × 10^−6^ to 1 × 10^−4^ mol L^−1^, and a measurement was performed after each addition. In this way, an excess of each lanthanide in relation to the Pb(II) content of 100 to 10,000 times is obtained in the solution. An identical series of measurements was performed separately for each lanthanide. For all lanthanides, a 1000-fold excess relative to the Pb(II) concentration did not affect the voltammetric signal of lead. At higher excesses, the lead peak gradually decreased. In the case of a 10,000-fold excess, lanthanide ions caused the following reductions in the lead peak: La(III) by 60%, Ce(III) by 55%, Pr(III) by 60%, Nd(III) by 40%, Eu(III) by 20%, Gd(III) by 40%, Dy(III) by 45% and Yb(III) by 20%. As shown, La(III) had the most interfering effect, and the voltammetric curves recorded in the presence of its various concentrations are shown in Figure 6.

### 3.5. Analytical Applications

**Figure 7:**
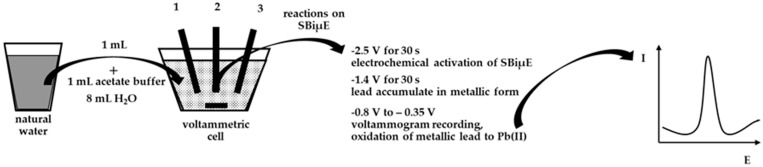
A schematic description of the procedure for analyzing natural water using SBiµE. 1—SBiµE working electrode. 2—Ag/AgCl reference electrode. 3—Pt counter electrode.

**Figure 8:**
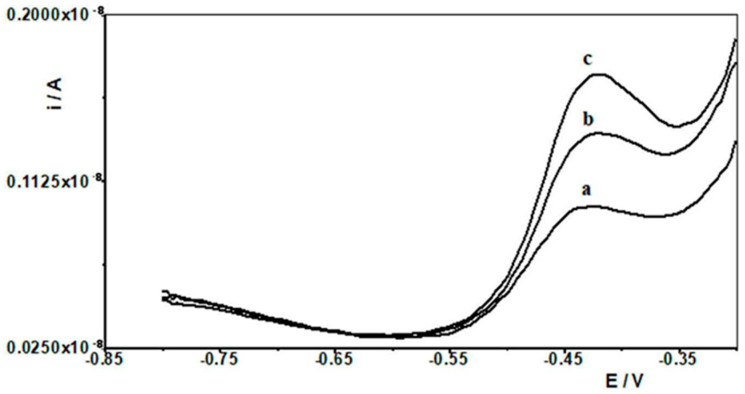
Differential pulse voltammograms obtained in the course of the Pb(II) determination in Bystrzyca river water: (a) Bystrzyca river water diluted ten times; (b) as (a) + 2.5 × 10^−10^ mol L^−1^ Pb(II); (c) as (a) + 5 × 10^−10^ mol L^−1^ Pb(II). Activation conditions −2.5 V and 30 s, and accumulation conditions −1.4 V and 30 s.

In order to evaluate the practical use of the developed DPASV procedure in combination with SBiµE as a working electrode, the determination of Pb(II) in aqueous environmental samples was carried out. A schematic description of the procedure for analyzing natural water is presented in Figure 7. In order to differentiate the samples for analysis, natural waters such as water from the Bystrzyca River and the Baltic Sea were selected. In both cases, the waters were stored after collection in the laboratory at approximately 6 °C. Analyses were carried out using the standard addition method after a 10-fold dilution of the analyzed water samples, and the results are presented in Table 2. The Baltic Sea contained Pb(II) in concentrations below the limit of quantification of the proposed procedure and was therefore spiked with different amounts of Pb(II), and recoveries were determined accordingly. Voltammograms obtained from the analysis of water from the Bystrzyca River are shown in Figure 8. The results of the analysis of real samples indicate that the proposed electrode was very accurate and precise and can be used for direct analysis of Pb(II) in real samples.

## 4. Conclusions

In this article, a new highly sensitive approach for the determination of trace Pb(II) concentrations based on the DPASV method in combination with a fixed solid bismuth microelectrode is described. Optimal determination conditions were optimized and determined. Using the activation potential of the electrode (−2.5 V) and the accumulation potential of lead (−1.4 V), it was demonstrated that the solid bismuth microelectrode enables the determination of extremely low lead concentrations, with a detection limit of 3.4 × 10^−11^ Pb(II). In addition to being very fast, the measurement is also very simple and only requires the addition of a supporting electrolyte, which was an acetate buffer, to the analyzed sample. During the analysis, it was not necessary to perform deoxygenation, which also simplifies the whole procedure. Compared to other voltammetric procedures for the determination of Pb(II) using a bismuth-based working electrode, our proposed procedure stands out for its exceptional sensitivity combined with good precision. The electrode used in this work is environmentally friendly in the laboratory and is part of the green chemistry trend. Finally, the proposed procedure was successfully applied to the analysis directly in aqueous environmental samples. Analyzing Pb(II) ions using our procedure is highly advantageous, low-cost, and simple to carry out.

## Figures and Tables

**Table 1:** An overview of working electrodes based on bismuth used for the determination of Pb(II) by the ASV method. The publications are ranked according to increasing limit of detection.

Working Electrode	Method	Linearity Range (mol L^−1^)	Detection Limit (mol L^−1^)	Application	Ref.
solid bismuth microelectrode	DP	1 × 10^−10^–3 × 10^−8^	3.4 × 10^−11^	river and sea water	this work
bismuth oxide surface-decorated nanoporous bismuth	SW	2.4 × 10^−9^–9.7 × 10^−7^	9.7 × 10^−11^	tap water	[50]
bismuth film multiwalled carbon nanotube Nafion composite modified glassy carbon electrode	SW	4.8 × 10^−9^–2.2 × 10^−7^	1.4 × 10^−10^	soil extract	[41]
gold nanoparticle-graphene-cysteine composite modified bismuth film glassy carbon electrode	SW	2.4 × 10^−9^–1.9 × 10^−7^	2.4 × 10^−10^	-	[45]
(BiO)_2_CO_3_ nanoparticles@SWCNT-Nafion/GCE	SW	ND–2.9 × 10^−7^	2.4 × 10^−10^	soil extract	[48]
bismuth film graphite carbon paste electrode	SW	4.8 × 10^−9^–3.4 × 10^−7^	3.8 × 10^−10^	soil extract	[40]
bismuth bulk electrode	SW	4.8 × 10^−8^–4.8 × 10^−7^	4.8 × 10^−10^	river water	[61]
nanocomposite functionalized multiwalled carbon nanotubes with polypyrrole film and bismuth particles carbon paste electrode	SW	5.3 × 10^−10^–5.8 × 10^−7^	4.8 × 10^−10^	tap water	[43]
bismuth film glassy carbon electrode with Zn mediator	DP	2 × 10^−9^–2 × 10^−6^	7 × 10^−10^	river water	[52]
solid bismuth microelectrode array	SW	2 × 10^−9^–2 × 10^−7^	8.0 × 10^−10^	lake and river water	[60]
polymer/bismuth film modified glass carbon electrode	SW	4.8 × 10^−9^–1.9 × 10^−7^	1.8 × 10^−9^	industrial wastewater	[44]
carbon paste electrode with BiOCl (bulk modifier)	SW	4.8 × 10^−8^–1.9 × 10^−6^	2.0 × 10^−9^	soil extract	[47]
nanoporous bismuth electrode	SW	4.8 × 10^−9^–1.9 × 10^−7^	7.2 × 10^−9^	tap water	[42]
bismuth film screen-printed gold electrode	SW	9 × 10^−8^–1.4 × 10^−6^	1.4 × 10^−8^	river and tap water	[39]
bismuth oxycarbide Nafion modified glassy carbon electrode	DP	ND–2.4 × 10^−7^	1.7 × 10^−8^	deionized water and tap water	[46]
bismuth-film electrode supported on pencil-lead graphite	DP	2.3 × 10^−7^–1.1 × 10^−6^	5.6 × 10^−8^	battery industry wastewater	[51]

**Table 2:** Analytical results of natural water samples analysis and recovery of Pb(II) added.

Sample	Pb(II) Added (nmol L^−1^)	Pb(II) Found (nmol L^−1^)	Recovery of Pb(II) (%)	RSD (%) *n* = 5
Bystrzyca River	-	0.24	-	6.2
Baltic Sea	2	1.87	83.5	5.5
	5	4.85	97.0	4.4
	10	9.75	97.5	4.6

## Data Availability

The original contributions presented in the study are included in the article; further inquiries can be directed to the corresponding author.
